# Association Between Periodontal Health Status and Pregnancy and Delivery Complications in Type 1 Diabetes Mellitus Pregnant Women: A Case-Control Study

**DOI:** 10.3290/j.ohpd.c_1789

**Published:** 2025-01-30

**Authors:** Matevž Janc, Marjeta Tomažič, Domen Kanduti, Uroš Skalerič, Rok Schara

**Affiliations:** a Matevž Janc Periodontist, IMJPerio, Private Peridontal Practice, Krško, Slovenia. Study conception and design, data collection and investigation, data analysis and curation, wrote the manuscript, reviewed and edited the manuscript, read and agreed to the published version of the manuscript.; b Marjeta Tomažič Internal Medicine Specialist, Division of Internal Medicine, University Medical Center Ljubljana, Ljubljana, Slovenia. Study conception and design, methodology, data collection and curation, investigation, reviewed and edited the manuscript, study supervision, project administration, read and agreed to the published version of the manuscript.; c Domen Kanduti Periodontist and Teaching Assistant, Department of Oral Diseases and Periodontology, Faculty of Medicine, University of Ljubljana; Department of Oral Diseases and Periodontology, University Medical Center Ljubljana, Ljubljana, Slovenia. Data curation, wrote the manuscript, reviewed and edited the manuscript, visualization, read and agreed to the published version of the manuscript.; d Uroš Skalerič Professor, Department of Oral Diseases and Periodontology. Faculty of Medicine, University of Ljubljana, Ljubljana, Slovenia. Study conception and design, methodology, data collection and investigation, project administration, read and agreed to the published version of the manuscript.; e Rok Schara Assistant Professor, Department of Oral Diseases and Periodontology. Faculty of Medicine, University of Ljubljana; Department of Oral Diseases and Periodontology, University Medical Center Ljubljana, Ljubljana, Slovenia. Study conception and design, methodology, data collection and investigation, data analysis, study supervision, project administration, read and agreed to the published version of the manuscript.

**Keywords:** C-section, gestational week of birth, periodontal disease, type 1 diabetes mellitus.

## Abstract

**Purpose:**

To assess the association between periodontal health and pregnancy or delivery complications in type 1 diabetic (TIDM) and non-diabetic pregnant women.

**Materials and Methods:**

15 TIDM and 15 non-diabetic primiparous women were enrolled in the prospective case-control study. We compared periodontal status, levels of glycosylated hemoglobin (HbA1c), gestational week of birth, birth weight of a newborn and pregnancy or delivery complications between the groups.

**Results:**

TIDM pregnant women gave birth statistically significantly earlier (2 weeks) (p = 0.034), but not before the 37th week of gestation. The odds ratio (OR) for pregnancy or delivery complications was ~ 5 times greater (95% CI: 1.1–26.4; p = 0.033) and for Caesarean section (C-section) ~ 6 times greater (95%CI: 1.2–30.7; p = 0.032) in TIDM group. The association between periodontal disease (PD) and pregnancy or delivery complications was not statistically significant in either group. The presence of TIDM (p = 0.002; R^2^ = 0.28), a higher bleeding-on-probing/full-mouth bleeding score (FMBS) (p = 0.043; R^2^ = 0.14), and a higher level of HbA1c (p = 0.026; R^2^ = 0.16) were statistically significantly more often associated with an earlier gestational week of birth. Higher levels of HbA1c were statistically significantly positively associated with a higher frequency of pregnancy or delivery complications (p = 0.024) and a higher frequency of C-section (p = 0.051).

**Conclusion:**

There are strong indications that both endocrinological and periodontal therapy should form a part of preventive prenatal care.

One of the major challenges of modern obstetrics is to prevent pre-term birth (between the 22nd and 37th week of gestation), as its incidence is slowly but steadily rising. Pre-term birth is the main reason of perinatal mortality and morbidity. 1.2% of pregnant women in Slovenia give birth very prematurely (before the 32nd week of gestation), and 5.5% between 32nd and 36th week of gestation.^44^ Because of the association between maternal inflammations (e.g., UTI) and pre-term birth and low birth weight in newborns, the hypothesis has been put forth on the association between the periodontal disease (PD) and complications during pregnancy and delivery. This has been evaluated in numerous studies.^18,21^ The increased risk of complications during pregnancy and delivery caused by periodontal disease is associated with increased levels of inflammatory mediators, such as TNFα, IL-6, IL-1β or PGE2, and/or toxins causing ectopic or systemic inflammatory reactions.^17,25,45^ The degradation of periodontal tissue and related complications in pregnancy are influenced by different factors, such as age, habits (e.g., smoking) and presence of systemic diseases during pregnancy (e.g., diabetes mellitus).^20^

## Periodontal Disease and Pregnancy

Periodontal disease (PD) is one of the most common chronic inflammatory diseases in humans,^27^ with a reported prevalence of 10–60% in adults.^11,26,49^ It is caused primarily by anaerobic, Gram-negative bacteria that colonise the subgingival area (bacterial plaque), which causes increase of pro-inflammatory cytokine release.^11^ The disease is clinically defined by the loss of periodontal tissue support, which is commonly assessed by radiographic bone loss or interproximal loss of clinical attachment measured by probing (commonly ≥ 4 mm with BOP), the number of teeth lost due to periodontitis, the number of teeth with intrabony lesions and the number of teeth with furcation lesions.^37^ Different risk factors also play an important role in an individual’s inflammatory response, such as smoking, poor oral hygiene, nutrition and stress.^9^ It has a complex correlation with different systemic diseases of inflammatory nature. Periodontal inflammation is closely linked to metabolic diseases (diabetes, obesity),^27,30^ cardiovascular diseases, arterial wall thickness and stiffness, endothelial dysfunction, dyslipidhemia and oxidative stress.^28,33^ Many studies have also indicated a link between the inflammation of the periodontal tissue and complications during pregnancy, such as pre-term birth (before the 37th week of gestation), low birth weight in newborns (≤ 2500 g), pre-eclampsia, which manifests itself as an increase in blood pressure (>140/90 mm Hg), and proteinuria (urinary proteins > 0.3 g/24 h) in pregnant women after the 20th week of gestation.^2,3,18,50^ The studies have also shown a correlation between the rise of the serum concentration of acute-phase C-reactive protein (CRP), an indicator of systemic inflammation, the increase in total periodontal inflammatory burden (TPIB),^37^ and pre-term births,^2,29,32^ pre-eclampsia^2,32,42^ and inhibited fetal growth during pregnancy.^2,43^

## Type 1 Diabetes Mellitus and Pregnancy

Maternal hyperglycemia resulting from type 1 diabetes mellitus at the time of conception substantially increases the probability of spontaneous miscarriage and morbidity and mortality of the mother and the fetus.^12^ Because type 1 diabetes mellitus (TIDM) pregnant women have a higher probability of pre-term birth, pre-eclampsia, macrosomia, distortion of the shoulder frame, intrauterine death, inhibited fetal growth, cardiac and renal malformations, it is necessary for TIDM women to plan their pregnancy and then have intensive glycemic control, as this reduces the likelihood of occurrence of those complications.^48^ Studies have shown that pre-eclampsia occurs in 12–15% of pregnant women with type 1 diabetes mellitus, while in only 5–7% of healthy pregnant women.^13,39^ Pregnant women with type 1 diabetes mellitus participating in the Lepercq et al study^23^ gave birth pre-term in 24% of cases, and the study confirmed a statistically significantly positive correlation between the spontaneous pre-term birth and the level of glycosylated hemoglobin (HbA1c). HbA1c is also being linked with fetal malformations, as pregnant women with a HbA1c level between 5% and 6% should have a normal pregnancy, while HbA1c levels of > 10.1% can lead to the occurrence of neonatal malformations in 20–25% of cases.^15^ Despite the increasingly stricter and better controlled blood glucose levels and the development of insulin treatment over the years, the prevalence of macrosomia in newborns of diabetic pregnant women remains constant^34^ and the likelihood of distortion of the shoulder frame during vaginal birth doubles when the weight of the fetus exceeds 4000 g.^1^

The aim of this study was to assess the association between periodontal health and pregnancy or delivery complications in type 1 diabetic (TIDM) and non-diabetic pregnant women. Primary measures of the study were clinical periodontal parameters, level of glycosylated hemoglobin and occurance of pre-eclampsia, pre-term birth, low birth weight and the need for C-section delivery. The study hypothesis was that TIDM primiparous women would have poorer periodontal health with higher blood levels of glycosylated hemoglobin, and were expected to have a higher incidence of pregnancy or delivery complications, such as pre-eclampsia, low birth weight in newborns, and a higher rate of C-section delivery.

### MATERIALS AND METHODS

#### Participants and Methods

The present investigation was part of the study entitled “Periodontal status of type 1 and type 2 diabetes mellitus pregnant women and of pregnant women without diabetes”, which was approved by the National Medical Ethics Committee (number 113/04/12) and performed in accordance with the ethical standards as laid down in the 1964 Declaration of Helsinki. All patients signed informed written consent to participate in the present study.

Thirty (30) primiparous women were included in the study: a “case group” of 15 primiparous women with type 1 diabetes mellitus and a “control group” of 15 healthy primiparous women. All participating women were younger than 35, non-smokers or former smokers who quit at least one year before conception, had not received periodontal treatment in the last six months, were not taking antibiotics three months before the start of pregnancy, were between the 28th and 34th week of gestation and had at least 20 of their own teeth, excluding wisdom teeth. Apart from type 1 diabetes mellitus in the case group, participants did not have any other systemic diseases. Only primiparous women who conceived naturally and were carrying singletons were enrolled in the study.

From the post-delivery reports, we obtained information on the birth details of the newborns, potential complications during pregnancy and delivery for all pregnant women. The data were confirmed through a phone interview with each participant. The permanent staff of the Gynecology Clinic of the University Medical Center in Ljubljana, who was not aware of the participation of the patients in our study, assisted the deliveries. For all participants, the gestational week was determined based on the last menstrual cycle and ultrasound examination in the first trimester.

The prevalence of periodontal disease depended on the selection of the criteria. The criteria for the dichotomous definition of periodontal disease from some similar studies ^4,5,6,19,20,35,36^ could not be used for the statistical analysis, since the prevalence of periodontal disease according to those criteria was either 0% or 100%. Only two criteria from two studies^7,16^ were usable. To determine the impact of periodontal disease on the occurrence of complications during pregnancy and delivery, we applied a periodontal pocket-depth criterion (≥ 4 sites with periodontal pocket depth ≥ 4 mm, as also done by Holbrook et al^42^) to define periodontal disease, but we measured the PPD on 6 sites per tooth and did a full-mouth examination.

Between the 28th and 34th week of gestation, a full-mouth periodontal examination was performed on all participating women and the following clinical parameters were assessed:

Full-mouth plaque score (FMPS) was calculated as the percentage of tooth surfaces with the presence of plaque at six sites per tooth. Plaque was detected using a periodontal probe;
Probing pocket depth (PPD) was measured from the gingival margin to the base of the clinical pocket using manual probe at six sites around each tooth;
Gingival recession (GR) was measured from the CEJ to the gingival margin using a manual probe at six sites around each tooth;
Clinical attachment level (CAL) was calculated as the distance in millimeters from the cemento-enamel junction (CEJ) or the cervical border of a restoration to the bottom of the probeable pocket at six sites per tooth. Two measurements were used to calculate the CAL: the PPD and the distance from the gingival margin to the CEJ;
Full-mouth bleeding score (FMBS) was measured at six sites around each tooth with the PPD measurements based on the presence or absence of bleeding up to 30 s after probing. The percentage of bleeding sites were calculated per subject per visit.

All measurements were performed by a single experienced clinician (MJ) at 6 sites per tooth and on all teeth present except third molars. A manual probe (Williams SE, PCP10, Hu-Friedy; Chicago, IL, USA) was used. Calibration sessions to measure the intra-examiner reliability showed a reproducibility of 93%.

Additionally, total bleeding periodontal wound (TBPW) size and total periodontal inflammatory burden (TPIB) were calculated.^38^

TBPW: This measures the average circumference of tooth cervices by tooth type, probing depth (PD) and bleeding on probing (BOP). The subgingival area for each tooth was calculated by multiplying 1/6th of the average cervical circumference of the tooth with each of the six measurements of probing depth. The sum of subgingival bleeding areas of all present teeth in one subject represented the total subgingival area of an individual.
TPIB: This measures of the average circumference of tooth cervices by tooth type, probing depth (PD) and bleeding on probing (BOP). The subgingival area for each tooth was calculated by multiplying 1/6th of the average cervical circumference of the tooth with each of the six measurements of probing depth. The sum of subgingival areas of all present teeth with PPD > 3 mm and all present teeth with positive BOP in a subject represented the total subgingival area of an individual.

From the medical records of participating women, glycosylated hemoglobin (HbA1c) test results were obtained.

#### Statistical Analysis

The data were statistically analysed using the software program IBM SPSS Statistics (IBM Statistical Package for Social Sciences Statistics, Version 22.0; IBM, Armonk, NY, USA). For the comparison of individual periodontal parameters, a bivariate logistic regression was performed. Based on the measured periodontal parameters, participants were dichotomously divided according to the presence of the periodontal disease, using criteria from the different studies.^26^ The odds ratio was defined with the 95% confidence interval (CI). Spearman’s correlation coefficient was used to test the impact of age, body mass index (BMI) and duration of TIDM on the individual periodontal parameters of primiparous women within their respective groups (test and control). A simple linear regression was used to measure the effects of age, body mass index (BMI), probing pocket depth (PPD), clinical attachment loss (CAL), bleeding on probing (FMBS), total periodontal inflammatory burden (TPIB), total periodontal bleeding wound (TPBW), glycosylated hemoglobin (HbA1c) and diabetes mellitus on birth weight (BW) in newborns and the gestational week of delivery (GWD). For all tests, statistical significance was set at p<0.05.

### RESULTS

**Fig 1:**
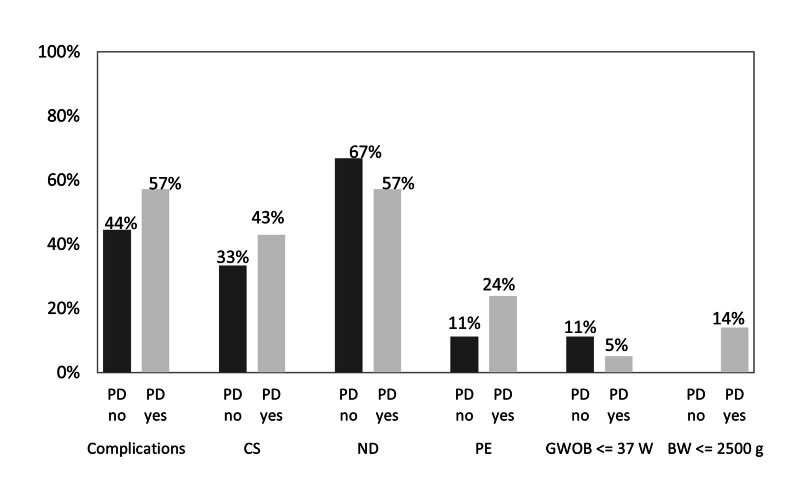
The incidence of complications during pregnancy and/or delivery in primiparous women with/without periodontal disease as a percentage; PD: periodontal disease; PE: preeclampsia; GWOB <= 37th W: gestational week of birth before the 37th week of gestation; BW <= 2500 g: birth weight of newborns less than 2500 g.

Pregnant women with poorer periodontal health had more frequent complications during pregnancy or delivery, but the association between the presence of periodontal disease and the complications was not statistically significant (Fig 1).

TIDM pregnant women had a 5.7-times (0.9; 34.5) higher probability of periodontal disease occuring compared to the healthy ones. The the odds ratio was marginally statistically non-significant (p = 0.059) (Table 1).

**Fig 2:**
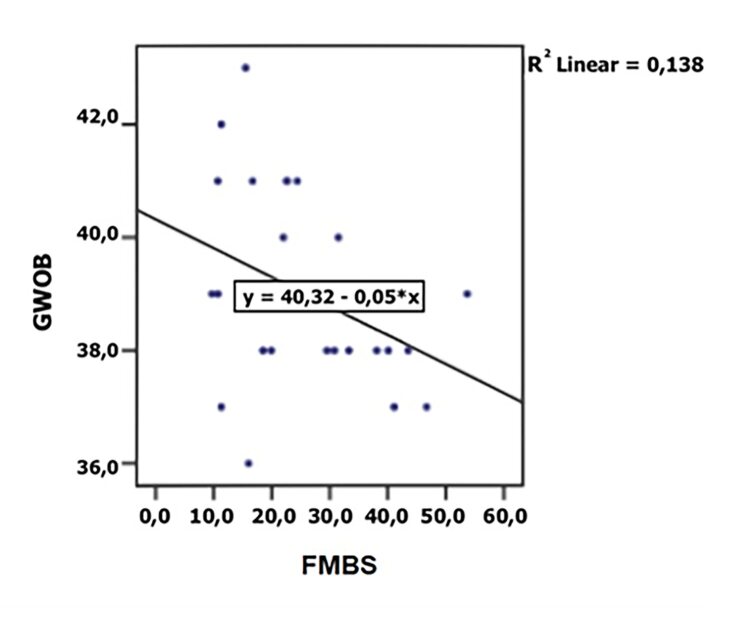
Association between the gestational week of birth (GWOB) and the full-mouth bleeding score (FMBS in %).

**Fig 3:**
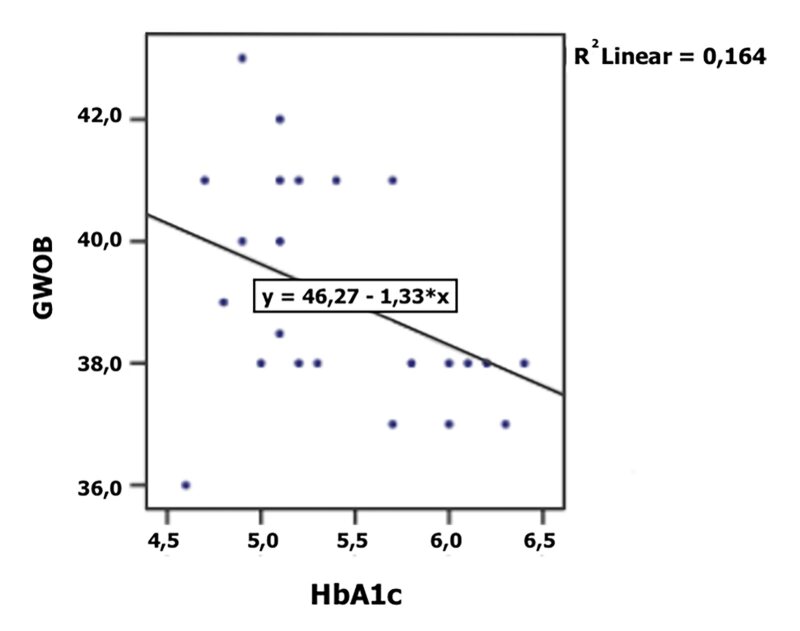
Association between the gestational week of birth (GWOB) and the level of glycosylated hemoglobin (HbA1c in %).

The test group of women delivered statistically significantly (2 weeks) earlier than the controls (p = 0.034), but not before the 37th week of gestation. The probability of complications during pregnancy or delivery was 5 times higher (95% CI: 1.1 – 26.4; p = 0.033), and the probability of a C-section delivery was 6 times higher (95% CI: 1.2–30.7; p = 0.032) in the TIDM group of pregnant women (Table 1). Type 1 diabetes mellitus (TIDM) (p = 0.002; R^2^ = 0.28), full-mouth bleeding score (FMBS) (p = 0.043; R^2^ = 0.14) and the level of glycosylated hemoglobin (HbA1c) (p = 0.026; R^2^ = 0.16) statistically significantly influenced the gestational age of birth (Table 2, Figs 2 and 3). The level of glycosylated hemoglobin (HbA1c) also had a statistically significant effect on the incidence of complications during pregnancy and delivery (p = 0.022) and the need for a C-section delivery (p = 0.011) (Table 3).

### DISCUSSION

The study has shown that the periodontal tissue of TIDM pregnant women is different from that of healthy pregnant women. The difference in terms of the prevalence of the periodontal disease within the group as well as between the groups depends on the definition of the periodontal disease, as recently shown by other studies.^26,44,47,49^ In this study, we used the 9 most frequently applied criteria in case-control studies. In our study, the prevalence of the periodontal disease in pregnant women, when applying the dichotomous criteria from studies by Jeffcoat et al,^19^ Jarjoura et al,^20^ Castaldi et al,^6^ and Bassani et al,^5^ was 100%; when applying the criteria used by Radnai et al,^33,36^ it was 95%; those of Cruz et al^7^ yielded 85%; Holbrook et al’s^16^ yielded 75%. In contrast, when applying the dichotomous criteria from the study by Bošnjak et al,^4^ the prevalence of the periodontal disease was 0%. These results demonstrate that pregnant women in Slovenia compared to pregnant women around the world suffer from more advanced periodontal disease. This was also confirmed by comparing the severity with which the periodontal tissue was affected (initial, moderate and advanced periodontal disease) with the observations of Bassani et al.^5^ In that study, 46.1% of pregnant women had periodontal disease in the initial stage, 13.8% of cases had periodontal disease in the moderate stage, and 2.2% advanced stage. In the present study, initial-stage periodontal disease was found in 35% of pregnant women, 55% had moderate periodontal disease, and in 10% of cases, the advanced stage was documented. The explanation for this difference lies in the fact that this study involved a smaller number of pregnant women, where half of pregnant women had type 1 diabetes mellitus with statistically significantly more affected periodontal tissue than healthy pregnant women, although the difference in prevalence of periodontal disease between the two groups was not statistically significant (p = 0.538 according to Cruz et al^7^; p = 0.147 according to Holbrook et al^16^). Compared to the study performed in our region,^4^ which included almost the same number of pregnant women with a periodontal disease prevalence of 3.2%, the periodontal status of the pregnant women in our study was better, as periodontal disease prevalence among these pregnant women was 0%, according to the criteria of that study.

The probability of developing periodontal disease was ≥ 5.7-times (0.9; 34.5) higher in the TIDM group of pregnant women compared to healthy pregnant women. The odds ratio was marginally statistically insignificant (p = 0.059). With a larger pool of pregnant women, this difference could have become statistically significant, but the results of our study nevertheless suggest that type 1 diabetes mellitus during pregnancy adversely affects the status of periodontal tissues, confirming the hypothesis put forward by other authors.^14^

The results of the correlation between TIDM and pregnancy and/or delivery complications in our study show that TIDM pregnant women had a statistically significantly higher probability (≥ 5-times; 95% CI: 1.1 – 26.4; p = 0.033) of experiencing pregnancy and/or delivery complications and a statistically significantly higher probability (≥ 6 times; 95% CI: 1.2 – 30.7; p = 0.032) of a C-section delivery than healthy pregnant women, which was confirmed in several other studies.^14,24,39,48^ In the study by Miailhe et al,^24^ emergency C-section deliveries among TIDM pregnant women were associated with increased glycosylated hemoglobin (HbA1c > 6.3%). Similarly, in our study, the level of HbA1c statistically significantly affected the occurrence of complications during pregnancy and/or delivery (p = 0.022) and the indication for C-section (p = 0.011); therefore, it is important to strictly control the level of blood sugar among TIDM pregnant women. In a healthy population, the level of HbA1c can be affected by body weight,^10^ which was also reflected in the group of healthy pregnant women in our study, but the association was only marginally statistically insignificant (p = 0.058).

Although there were more complications during pregnancy and/or delivery among women with periodontal disease, after adjusting the odds ratio for age, smoking, and the presence of TIDM, periodontal disease did not have a statistically significant correlation with complications during pregnancy and/or delivery (p = 0.108), which corresponds to the findings of Holbrook et al.^16^ Due to the small sample size, we cannot exclude the possibility of randomness in the sample, which could limit the interpretation of the results. However, the results of our study are representative for the population of pregnant women in Slovenia, as all non-smoking primiparous women with type 1 diabetes mellitus were included for the duration of the study. For the 80% standard power of the test at such prevalence and variance of periodontal disease, a sample size of 187 pregnant women would be necessary, which implied that the study should last for at least 10 years in order to obtain a sufficient number of pregnant women with TIDM in Slovenia.

As independent variables, TIDM (p = 0.002, R^2^ = 0.28), FMBS (p = 0.043, R^2^ = 0.14) HbA1c (p = 0.026, R^2^ = 0.16) had a statistically significant correlation with an earlier GWOB. TIDM pregnant women in our study gave birth statistically significantly (2 weeks) earlier than healthy pregnant women (p = 0.034), which can be explained by a statistically significantly higher level of HbA1c (p = 0.003). Despite earlier GWOB, TIDM pregnant women did not deliver too early (before the 37th week of gestation), which can be attributed to the good control of the level of glycosylated hemoglobin (HbA1c = 5.8%). The correlation between HbA1c and GWOB in our sample implied that the level of HbA1c > 7% can pose a risk for pre-term birth (before the 37th week of gestation), while the level of HbA1c > 10.7% can lead to a very pre-term birth (before the 32nd week of gestation). Similar findings were established in some other studies.^15,40,51^

The correlation between FMBS and GWOB was also confirmed by some other studies,^11,22^ but the results of our study also indicate that 67% or more of BOP sites can pose a risk for a pre-term birth.

Because of a small sample of enrolled pregnant women and a statistically significant relation between HbA1c and TIDM, the multiple linear regression (see Stevens^41^) performed here only included TIDM and FMBS. The test showed a statistically significant correlation (p = 0.022) between TIDM and GWOB, while FMBS did not have a statistically significant impact (p = 0.425) on GWOB.

In this study, the incidence of pre-eclampsia in TIDM pregnant women was slightly higher (33%) than in some other studies (12–15%),^13,39^ with the incidence in healthy pregnant women (6.7%) comparable to the results of the above-mentioned studies (5–7%). More frequent pre-eclampsia in this study’s test group can also be due to the limited sample size. The incidence of pre-term birth in our survey was 20%, which is only slightly lower than in the study by Lepercq et al,^23^ where it was 24%. None of the observed factors had a statistically significant impact on the birth weight of the newborn.

Another limitation of the present study is its case-control study design. A cross-sectional or cohort study to group the mothers and measure the outcomes according to periodontal status and type of diabetes or non-diabetes would allow the interaction of the effect of diabetes with periodontal status and pregnancy outcomes to be evaluated. Further research is required to investigate the correlation of periodontitis, different systemic conditions of pregnant women, and pregnancy or delivery complications.

### CONCLUSION

There are strong indications that both endocrinological and periodontal therapy should form a part of preventive prenatal care, especially in cases of type 1 diabetes in the mother.

**Table 1:** Pregnancy and/or delivery complications and periodontal disease in TIDM pregnant women (test group) and healthy pregnant women (control group)

**Complications**					
no	14 (46.7)	10 (66.7)	4 (26.7)		
yes	16 (53.3)	5 (33.3)	11 (73.3)	5.5 (1.1; 26.4)	0.033
**C-section**					
yes	18 (60)	12 (80)	6 (40)		
no	12 (40)	3 (20)	9 (60)	6 (1.2; 30.7)	0.032
**Pre-eclampsia**					
no	24 (80)	14 (93.3)	10 (66.7)		
yes	6 (20)	1 (6.7)	5 (33.3)	7 (0.7; 69.5)	0.097
**Pre-term birth** GWOB < 37th week
no	28 (93.3)	14 (93.3)	14 (93.3)		
yes	2 (6.7)	1 (6.7)	1 (6.7)	1 (0.1; 17.6)	1
GWOB	39 (39; 1.6)	39.9 (40; 1.8)	38.2 (38; 0.8)	0.4 (0.2; 0.8)	0.013
**Birth weight < 2500 g**
no	27 (90)	14 (93.3)	13 (86.7)		
yes	3 (10)	1 (6.7)	2 (13.3)	2.1 (0.2; 26.7)	0.55
Birth weight	3.4 (3.4; 0.5)	3.4 (3.4; 0.4)	3.4 (3.3; 0.6)	1.04 (0.3; 4.2)	0.950
**Periodontal disease**
no	9 (30)	7 (46.7)	2 (13.3)		
yes	21 (70)	8 (53.3)	13 (86.7)	5.7 (0.9; 34.5)	0.059

**Table 2:** Association between different risk factors and gestational week of birth (GWOB), results of simple linear regression

Age	0.01	-0.1	0.594
Body mass index (BMI)	0.01	-0.11	0.557
Probing pocket depth (PPD)	0.00	-0.06	0.740
Clinical attachment loss (CAL)	0.00	-0.05	0.804
Full-mouth bleeding score (FMBS)	0.14	-0.37	0.043
Total periodontal inflammatory burden (TPIB)	0.00	0.00	0.986
Total bleeding periodontal wound (TBPW)	0.05	-0.23	0.217
Type 1 diabetes mellitus (TIDM)	0.28	-0.53	0.002
Glycosylated haemoglobin (HbA1c)	0.16	-0.41	0.026
Periodontal disease (PD)	0.90	-0.30	0.108
R^2^: coefficient of determination; Std.B: standardised regression coefficient.

**Table 3:** Univariate OR for glycosylated hemoglobin (HbA1c) as a risk factor for pregnancy and/or delivery complications (univariate logistic regression test), values presented as mean (median, standard deviation)

HbA1c	5.2 (5.1; 0.4)	5.6 (5.7; 0.5)	9.7 (1.4; 67.2)	0.022
	C-section no	C-section yes	OR (95 % CI)	p-value
HbA1c	5.2 (5.1; 0.5)	5.7 (5.7; 0.4)	14.9 (1.9; 119)	0.011
OR: odds ratio; CI: confidence interval.
